# Novel Method for the Manufacture of Complex CFRP Parts Using FDM-based Molds

**DOI:** 10.3390/polym12102220

**Published:** 2020-09-27

**Authors:** Paul Bere, Calin Neamtu, Razvan Udroiu

**Affiliations:** 1 Department of Manufacturing Engineering, Faculty of Machine Building, Technical University of Cluj-Napoca, Memorandumului 28, 400114 Cluj-Napoca, Romania; 2 Department of Design Engineering and Robotics, Faculty of Machine Building, Technical University of Cluj-Napoca, Memorandumului 28, 400114 Cluj-Napoca, Romania; 3 Department of Manufacturing Engineering, Faculty of Technological Engineering and Industrial Management, Transilvania University of Braşov, Mihai Viteazu 5, 500174 Braşov, Romania

**Keywords:** CFRP, PLA mold, additive manufacturing, fused deposition modeling, vacuum bag technology, 3D scanning, bike saddle

## Abstract

Fibre-reinforced polymers (FRP) have attracted much interest within many industrial fields where the use of 3D printed molds can provide significant cost and time savings in the production of composite tooling. Within this paper, a novel method for the manufacture of complex-shaped FRP parts has been proposed. This paper features a new design of bike saddle, which was manufactured through the use of molds created by fused deposition modeling (FDM), of which two 3D printable materials were selected, polylactic acid (PLA) and acrylonitrile butadiene styrene (ABS), and these molds were then chemically and thermally treated. The novel bike saddles were fabricated using carbon fiber-reinforced polymer (CFRP), by vacuum bag technology and oven curing, utilizing additive manufactured (AM) molds. Following manufacture the molded parts were subjected to a quality inspection, using non-contact three-dimensional (3D) scanning techniques, where the results were then statistically analyzed. The statistically analyzed results state that the main deviations between the CAD model and the manufactured CFRP parts were within the range of ±1 mm. Additionally, the weight of the upper part of the saddles was found to be 42 grams. The novel method is primarily intended to be used for customized products using CFRPs.

## 1. Introduction

Carbon fiber-reinforced polymers (CFRPs) often offer greater advantages than most other commonly used materials and are frequently used in many fields such as the aerospace, automotive, railway, naval, sports industry, medical, and civil construction industries [1,2]. Within the sports industry (CFRP bicycles, CFRP tennis rackets etc.) due to their excellent physical and mechanical properties, such as being lightweight, high strength, etc., CFRP products offer a good opportunity to improve the performance of the participants.

The uses of polymers are very popular within additive manufacturing (AM) processes having applications across many domains [3]. Fused deposition modeling (FDM) or fused filament fabrication (FFF) is defined as a material extrusion process within ISO/ASTM 52900:2015 standard [4]. Recently, due to the low production costs and the ability to feature a high degree of automation, the technology surrounding FDM/FFF has developed in a relatively rapid fashion [5,6,7,8]. This technology allows for the manufacture of complex parts, at a relatively low production cost, from various thermoplastic filaments including polylactic acid (PLA), acrylonitrile butadiene styrene (ABS), polycarbonates, nylons, etc. However, there are some limitations to the FDM process, for example the thermoplastic parts have poor mechanical properties when compared to traditional manufacturing methods, such as injection molding [9]. PLA is often treated with more importance in FDM, over other comparable materials, due to its relatively low production cost, good three-dimensional (3D) printability, and biodegradability; in addition to this, PLA has a low thermal tension, which reduces warping during the FDM process [10]. ABS material contains three monomers: acrylonitrile (A) in a proportion of 15%, butadiene, (B) 35% and styrene, (S) 60%. It should also be noted that ABS has good thermal and mechanical properties, resulting in ABS being used to produce jigs, fixtures, and tooling. The styrene from this material contributes to the achievement of a shiny surface. ABS is also compatible with soluble support materials for easy support removal, allowing more complex prints to occur.

Post-processes methods and variation of different printing parameters can enhance mechanical properties and surface finish of the part made by FDM. Porter et al. have investigated the optimal infill percentage of the beams from PLA that can maximised specific flexural rigidity, and obtained an infill percentage in the range of 10% to 20% [11].The post chemical treatment using acetone (99%) and dichloroethane (98%) solutions dramatically improves the surface finish and dimensional accuracy, and reduce the tensile strength of FDM specimens made of ABS [12]. Several researchers have analysed the effect of different annealing times and temperatures on thermoplastic materials which was 3D printed by FDM/FFF techniques [13,14,15]. The results showed that annealed PLA shape memory polymer at 75 °C increased the tensile and compressive strength (6% increases in ultimate compressive strength) compared to as-printed samples [13]. Wach et al., have printed PLA specimens by the FDM process and annealed they over its glass transition temperature determined an increase of 11–17% in the flexural stress of the parts [15].

**Figure 1:**
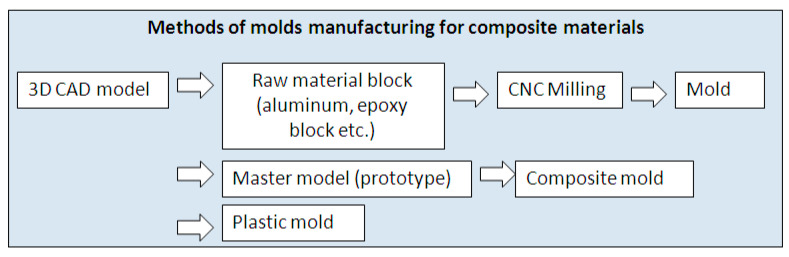
Methods of mold manufacturing for composite materials.

There is a great diversity of available manufacturing methods and processes for the production of composite material (CM) parts. Parts with a complex geometry often require the use of molds, which can be manufactured using various methods and technologies (Figure 1). Traditionally, molds are made by computer numerical control (CNC) milling a block of raw material or of various epoxy resins, of which, this technological process is time and manpower intensive, usually with high associated costs. An additional method of making molds is by using CM, where it is necessary to manufacture a master model by a separate method, tailored to the composite material the mold is made from. This process involves the deposition of FRP layers on to the part model, followed by the polymerization of the manufactured part then extraction of the mold from the master model. This process has a relatively high level of accuracy, however, it is time intensive.

Rapid tooling (RT) [8,10,16,17] is a technology designed for short manufacturing runs that adopt rapid prototyping (RP) 3D printing techniques and apply the techniques to making soft or hard tooling, determined by the material used. RT can produce tooling and molds indirectly, through use of a master 3D printed model, or directly though AM. Thus, the use of AM allows for a direct and rapid way to produce hard tooling, jigs, and molds from different strong materials of the designer’s choice (metals, resins). A mold was manufactured by Udroiu, using 3D inkjet printing techniques, fabricated out of the following materials: ZP 131 composite powder, ZB60 binder, and Z-max epoxy resin [16]. The mold was then used for the manufacturing of a part for an aircraft joystick, comprised of rubber. A research study of acrylic thermoplastic molds based on polymer jetting technology concluded that 3D printing is a rapid manufacturing method which can be used in a small series production of parts by using reaction injection molding (RIM) and casting [17]. Rodriguez et al. investigated the use of FDM technology to create molds using various types of material, for the thermoforming process [18], and found that the properties of the mold can transfer the surface defects of FDM technology to the thermoformed material sheets. Compression molds were 3D-printed using PLA and ABS filaments and then polished to produce personalized dental fillers [19]; this study demonstrated that 3D printed molds are able to be used in dental applications. Hay et al. used a FDM-printed mold of PLA to cast a customized PMMA (polymethyl methacrylate) implant for an external surface of a cranium [20]. Sieminsky et al. analyzed FDM/FFF technology used to manufacture mold inserts from PLA [21], to increase the life of the inserts and reduce the production time cycle, the inserts should be cooled using compressed air, after the removal of the molded part. Lusic et al. has analyzed, through the use of FEM (finite element method) simulations, the potential of CFRP-laminating molds to be used as rapid tooling through use of FDM [22]. It was concluded that thermoplastic molds comprising ULTEM 1010 material, manufactured by FDM, could be used for the manufacturing of CFRP components in autoclaves; however, they did not validate their FEM results. Additionally, Sudbury et al. investigated an industrial-grade approach to additive manufacturing, similar to extant FDM process used to produce molds used for hand lay-up of the fiberglass part [23]. The 3D-printed molds were machined by a CNC mill to improve the quality of the finished surface, and this paper concluded that 3D printed molds could be an effective approach for limited production runs of composite parts, although it must be noted that the molds used in this paper were not exposed to temperature or pressure.

The reinforcement of fibers on the thermoplastic filaments used within the 3D printing process can be created by combining short fibers and continuous fibers. The rapid manufacturing process of CFRP using FDM technology is treated in [24]. The thermoplastic matrix and the CFRP powder is used in this case and the process parameters and mechanical properties are investigated. Zhang et al. proposed a method for the 3D printing of continuous carbon fiber-reinforced plastics with a pressure roller, and found that there was an improvement in the strength of parts printed as compared to traditional FDM parts; however, this printer is considered in an experimental stage and thus cannot be used for 3D printing of molds at present [25]. Nanoparticles and short or long fibers in the thermoplastic filament can increase the specific desired mechanical properties of PLA, ABS, nylon etc. [26,27,28], although the 3D printing of continuous fiber-reinforced composites manufactured through an FDM process is a relatively new technique still in its infancy; thus there is a lack of experimental data on the mechanical performance of structures manufactured using this process [26]. The internal defects [29,30,31] resulting from the FDM process decrease the desired mechanical properties of the specified thermoplastic-reinforced fibers, and they cannot be compared with traditional FRP. Thus, traditional fiber-reinforced plastic materials with light weight and high strength are often the best option to manufacture parts to improve the performances of the athletes.

At present, the measurement of tools and molds through optical 3D scanning has gained much interest in the quality control process for different processes, e.g., forging [32,33], injection molding [34], and CFRP parts [35].

The use of advanced materials within performance cycling has led to outstanding results, and specialist bicycle components are now becoming lighter whilst retaining desired special mechanical properties. Liu et al. proposed to manufacture a bike frame comprising CFRP, using finite element analysis (FEA) the stacking sequence of the layers was investigated, however, they did not validate the results of the FEA [36]. The use of high-performance MC, especially CFRP, through the preferential arrangement of the layers can lead to both low total end mass and desired mechanical properties. Depending on the individual, some cyclists require more rigid parts, so that as much energy as possible is transmitted directly to the pedals. Alternatively, some solutions are centered around more elastic elements, in order to dampen vibrations encountered through use. It is accepted that elements made of CFRP can satisfy both of the aforementioned main requirements, through distinct approaches and the correct orientation of the material filaments for preferential reinforcement can lead to spectacular results. The position of the body on the bike during seated sprint cycling is studied in [36,37,38,39]. The optimization of lower limb joint cinematics of the participants under maximal intensity cycling was investigated. Also, a bicycle saddle 3D printed in colors can shown simulated pressure distribution of a rider [40]. A bicycle saddle with a special shape was tested by Piazza et al., where the effects on perineal compression, blood perfusion, genital sensation and sexual function of the cyclist were investigated [41]. A manufacturing method for a bicycle saddle was presented in [42]; this was created based upon a master 3D printed model of the bike saddle manufactured from ABS material. Using the ABS master model, the authors have applied a traditional technology by hand layup to manufacture the mold from glass fiber and epoxy material. The accuracy of the parts obtained and this traditional mold was not studied.

Traditional molds (metal or epoxy resin block, composite molds), used for composite materials are often very robust; however, they are only cost-effective when used in conjunction with mass production. For production of small batches or customized parts, there is a growing need for molds which can be produced quickly and at a relatively low cost. 3D printing through the use of FDM/FFF processes, utilizing low-price filaments, such as PLA or ABS, could be an approach that will significantly reduce production time and costs to produce molds for CM.

In this paper, the authors propose a novel method to obtain a mold comprising a thermoplastic material, used for laminating the CFRP parts, using an additive manufacturing technique. Two materials were selected for evaluation, PLA and ABS, which were used to create the mold(s) using FDM. The design of the novel mold and CFRP bike saddle were then presented within this paper, where the newly manufactured CFRP parts and mold(s) were then scanned using non-contact 3D scanning methods in order to perform dimensional evaluation of the results; these results were then presented and compared against the original CAD model. It was found that the manufacturing technology used to create the mold and CFRP parts allowed for good results to be achieved.

## 2. Materials and Methods

### 2.1. Design of a Novel Bike Saddle and its Mold

**Figure 2:**
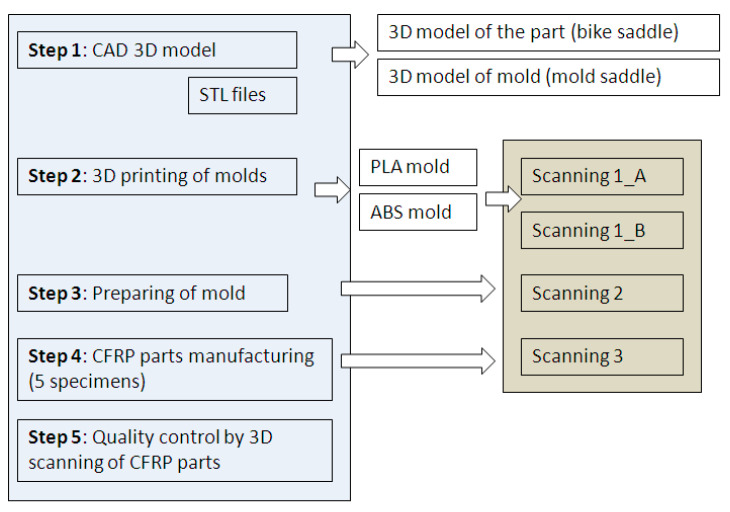
Novel method for manufacturing complex carbon fiber-reinforced polymer (CFRP) parts.

The proposed method consists of several steps as shown in Figure 2. The 3D CAD model of the mold is converted to a STL (standard triangulation language) file format. Within the second phase of the method, the mold is 3D printed by FDM using two different materials on two different 3D printers. The molds are prepared for vacuum bag forming methodology and oven curing, within the third step. Following the manufacture of CFRP, specimens are subjected to a quality inspection, using non-contact 3D scanning techniques, in order to perform the dimensional evaluation of the results. Three 3D scans were performed as follows for the 3D printed mold, the final mold, and the CFRP parts.

Saddles’ dimensions used worldwide for performance bicycles are in the range of 110–170 mm in width and 240–280 mm in length, respectively, depending on the type of bike, kind of competition, and individual customization. Chen investigated the measurement of the external ischial tuberosity width and the anthropometric data using a group of men and women to determine the bicycle-seat sizes [43]. The proposed predictive model assists riders to determine their seats size for bicycling.

**Figure 3:**
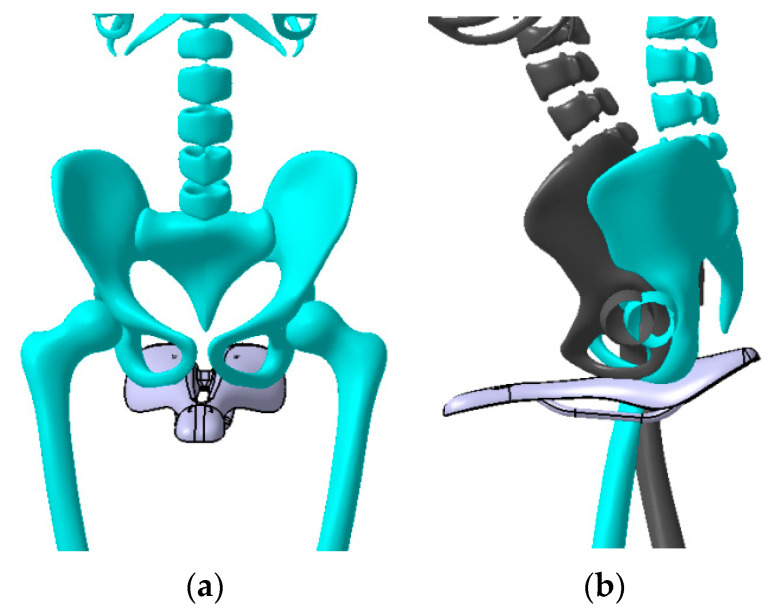
Determination of the design curves based on the human bones position on the bike saddle: (**a**) front view; (**b**) side view.

**Figure 4:**
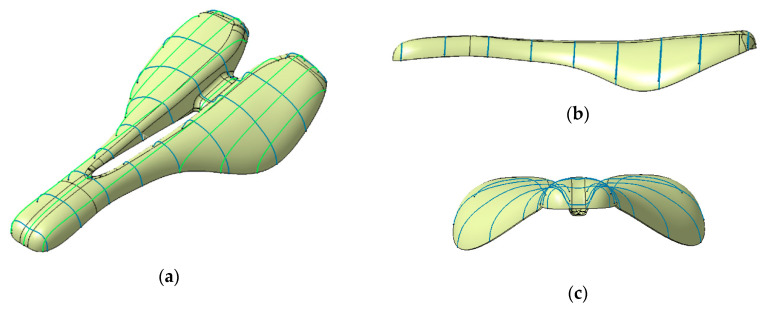
The CAD model of the novel saddle: (**a**) isometric view; (**b**) side view; (**c**) front view.

**Figure 5:**
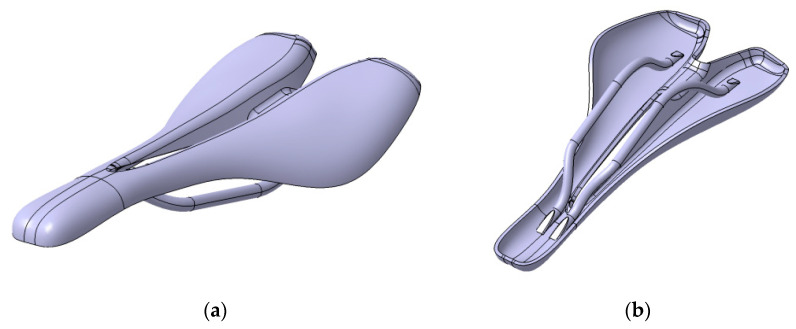
The 3D model of the assembled saddle: (**a**) upper side; (**b**) lower side.

A saddle with a width of 145 mm and a length of 270 mm was taken into consideration in this research. The surface area of the bike saddle is 3300 mm^2^. The design constraints of the saddle shape were established by analysis of the lateral and longitudinal position of the cyclist on the bicycle and by studying the anatomical particularities of the ischial bones (pelvic bones) and the perineum area of a group of cyclists (Figure 3). The authors cannot provide exact details on the anatomical dimensions of the analyzed individuals for reasons of protecting individual data. The geometric shape of the saddle (Figure 4) was determined taking into account the best possible contact of the pelvic bones with the saddle surface. A longitudinal channel designed on the central part of the saddle reduces the pressure applied in the perineum area on the prostate of male cyclists. Two CFRP bars of 8 mm in diameter are glued on the lower surface of the saddle, allowing the assembly to link with the bar inserted into the frame of the bike (Figure 5).

**Figure 6:**
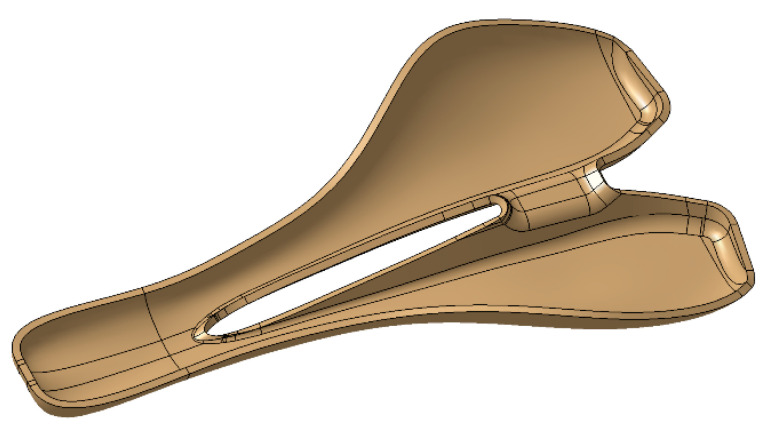
The 3D model of the mold saddle.

The 3D model of the saddle was designed based on non-uniform rational B-spline (NURBS) curves, and the multi-section surface technique within the CATIA V5 software (Dassault Systems, USA), as is shown in Figure 4a–c. A CAD model of the saddle mold with 4 mm wall thickness was obtained based on the 3D model of the saddle (Figure 6).

### 2.2. Manufacturing Method Using 3D Printing by Fused Deposition Modeling (FDM) Technology of the Mold Bike Saddle

**Figure 7:**
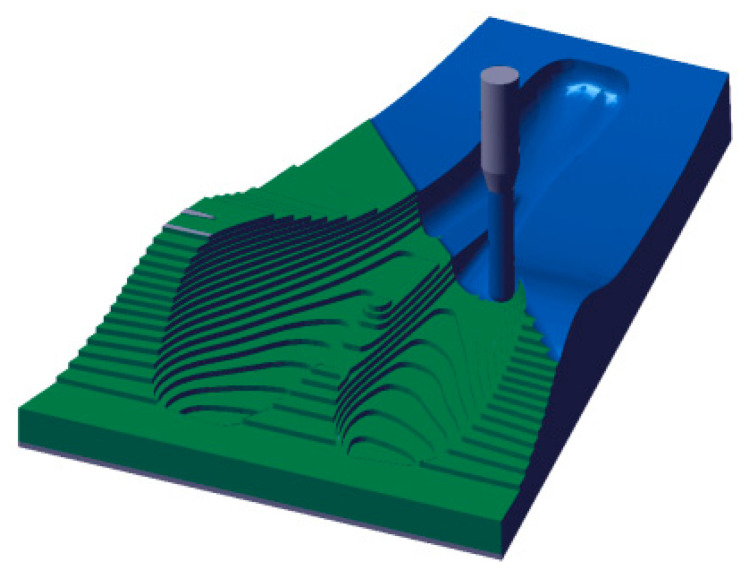
Mold manufacturing simulation by CNC milling.

Initially, a comparative study between two different mold manufacturing techniques was performed. Firstly, CNC milling using two different material blocks, aluminum and epoxy was considered. The processing time, the cost, and the masses of the mold were estimated by simulation. Two technological operations, roughing with a 6 mm end mill and finishing with a 3 mm ball-nosed tool were considered for manufacturing simulation within CATIA software. The mold manufacturing simulation by CNC milling is presented in Figure 7.

The new method of manufacturing molds involves making them from plastic materials by 3D printing. Two thermoplastic molds were 3D printed using two different printers. Thus, the first mold marked with ‘A’ was 3D printed from SMARTFIL PLA filament (Smart Materials 3D, Alcalá la Real, Spain) on the Leapfrog Creatr XL printer (Leapfrog 3D Printers, Alphen aan den Rijn, The Netherlands) and the second one marked with ‘B’ from ABS-M30 (Stratasys, Eden Prairie, MN, USA) on a STRATASYS Fortus 380 mc dual extruder printer (Stratasys, Eden Prairie, MN, USA). The diameter of each type of filament was 1.75 ± 0.05 mm. The properties of printed materials provided by the suppliers are presented in Table 1 [44,45] and the processing parameters of the 3D printing are shown in Table 2. The mechanical properties of FDM parts from PLA and ABS were investigated by many authors in different printing conditions [11,14,15,24,27,28,46,47].

**Figure 8:**
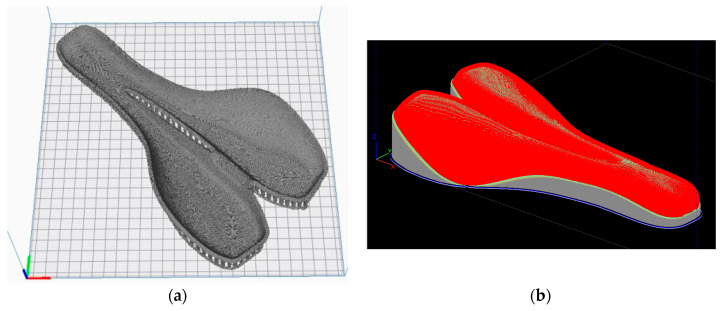
The 3D printing simulation of the molds: (**a**) mold ‘A’ made of PLA on Leapfrog Creatr XL; (**b**) mold ‘B’ made of ABS on STRATASYS Fortus 380 mc.

The STL file of the mold, saved from CATIA V5 software, was imported to every specific software of the two printers. This was used to generate G-code and 3D print settings. In the first study, Ultimaker Cura software [48] was used to generate a G-code file for mold A. The mold A was positioned with the functional surface upward on the build plate (Figure 8a). The mold B was preprocessed using the Insight 3D printing software (Stratasys, Eden Prairie, MN, USA). The build orientation of the part was optimized in diagonal position with the functional surface downward (Figure 8b). The support structure of PLA-based mold was mechanically removed in the post-processing stage. The SR30 soluble support material used for 3D printing of the ABS-based mold was removed by dissolving it in a very mild NaOH solution.

The surface roughness of the mold has been measured using a Namicon TR-220 (Namicon Testing S.R.L., Otopeni, Romania) roughness tester, as per the ISO 4288 standard [49]. The roughness was measured perpendicular to the deposition direction, following a measurement procedure similar as in [50]. As is concluded in [51] the surface obtained by FDM technology is the surface composed of high peaks. The surface roughness of the FDM-based mold is not appropriate for laminating and polymerizing CFRP. The active surface of the mold requires mechanical processing and deposition of filler materials for its uniformity. Thus, layers of polyester gel coat were applied and then polished to obtain a glassy surface.

The PLA material does not make a very good chemical connection with the polyester gel coat. A chemical surface treatment or a deposition of an intermediate layer of another material would be suitable. The surface of the PLA-based mold was initially treated additionally with a layer of epoxy gel coat. Thus, OH 38 hardener and CH-3 epoxy gel coat (Ebalta Kunststoff GmbH, Rothenburg o.d.t. Germany) that contains aluminium (Al) powder was used, where the mixing ratio is 100: 35 parts by weight. The treated PLA mold was polymerized for 24 h at 20 °C, and then T35 polyester gel coat (Havel Composites CZ s.r.o., Czech Republic) layer was applied by spraying on both sides; 2% methyl ethyl ketone peroxide (MEKP) catalyst was used to polymerize it. The epoxy layer allows a better adhesion of the polyester layer to the surface of the mold, and creates a good interface between the T35 gel coat layer and the PLA. Also, Al powder creates high stiffness properties of the mold. The covered layers were applied on both sides of the molds.

**Figure 9:**
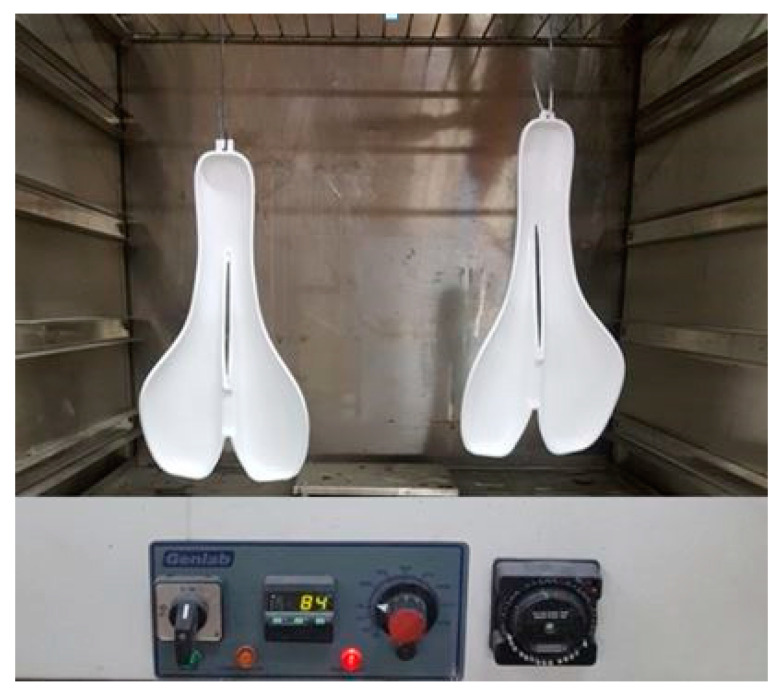
Heat treatment of the molds in the oven.

The ABS mold was sprayed with T35 gel coat layers. Also, the styrene from ABS allows a good chemical adhesion with the styrene component from the polyester gel coat. A heat treatment in the oven at a temperature of 85 ± 2 °C for 24 h was applied for both molds in order to prepare them for the lamination of CFRP layers (Figure 9). Finally, the PLA mold was mechanically processed with abrasive paper up to 1000 grids and polished.

### 2.3. Manufacturing Method of Carbon Fiber-Reinforced Polymer (CFRP) Bike Saddle

The CFRP bike saddle specimens were manufactured by vacuum bag technology and oven curing. The used prepreg materials are codified as GG245T-DT806W-42 (Delta Tech S.p.A., Rifoglieto Italy) and consist of carbon fiber Twill prepreg fabric type 2 × 2 by 245 g/sq, (245T), 3K threads, and epoxy resin type DT806W. This prepreg material contains 42 wfr. epoxy resin with a curing range from 65 °C to 140 °C.

The mold was prepared by applying mold sealer layers, and mold release layers that prevent the adhesion of the CFRP material at the mold surface. A layer of mold sealer type S31 (Jost Chemicals, Laudenbach, Germany) was applied in order to close the material pores and create a sleek surface. Then, the surface was treated with five layers of liquid mold release type Frekote 770NC (Loctite, Düsseldorf, Germany). Each layer was applied at 20 min interval to allow evaporation and drying of the solvent.

**Figure 10:**
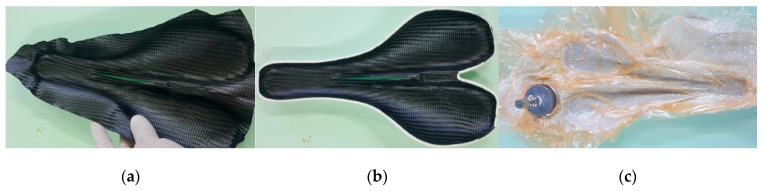
Application of CFRP prepreg layers on the PLA mold: (**a**) applying of a piece of CFRP prepreg; (**b**) cutting the edges of the CFRPs prepreg according to the PLA mold shape; (**c**) mold and composite material in the bag under vacuum pressure.

Three CFRP prepreg layers were applied on the mold surface following a stacking sequence distributions of [0/90/±45/0/90] degrees (Figure 10a). The edges of the CFRPs prepreg were cut according to the PLA mold shape (Figure 10b), and the whole assembly was covered with release foil and the breather fabric (Figure 10c). The mold was inserted into a vacuum bag which was sealed on the separation plane using an electric welding machine. A uniform pressure of −0.09 MPa was applied for 30 min on the composite surface. Then, the vacuum pump was stopped in order to check the bag tightness. The composite material was cured in an oven at 80 ± 20 °C for 5 h, where the vacuum pressure was applied.

The mold was cooled down for 30 min. The auxiliary materials have been removed (vacuum bag, the breather fabric, and the release foil) and the CFRP bike saddle was released from the mold. The CFRP part was then sanded on the borders by glass paper and the central longitudinal channel of the part was cut.

Five specimens denoted by A1-A5 from CFRP and two specimens denoted by C1-C2 carbon-Kevlar fiber-reinforced polymer (CKFRP) were manufactured using the PLA mold. The mass of all samples was determined using a precision scale.

### 2.4. Quality Control by 3D Scanning Technique of Molds and CFRP Parts

The molds and parts were subjected to a quality inspection, using non-contact 3D scanning techniques. The Creaform GO!Scan 20 (Creaform, Québec, Canada) 3D optical scanning system used in this study integrated with VXelements software (Creaform, Québec, Canada) provides a fast acquisition speed at 550,000 points/second, and extensive capture of complex surfaces at 0.1 mm accuracy.

**Figure 11:**
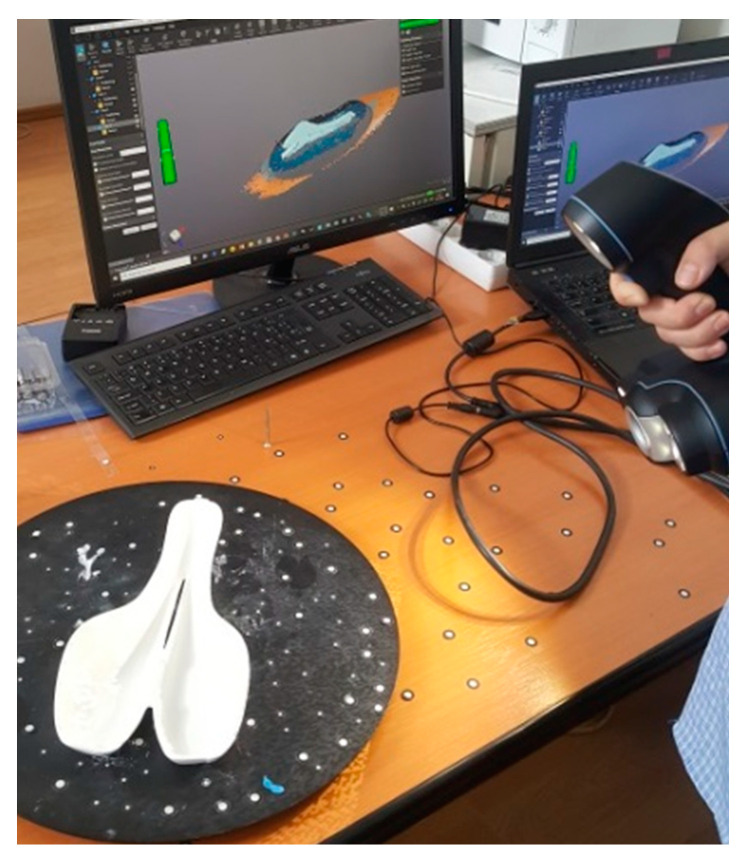
Positioning of the bike saddle on the rotary table of the scanning system

The parts sprayed with white powder were positioned on a rotary table on which positioning targets were placed (Figure 11). The scan was performed in one go resulting in a 3D mesh of points. VXelements software was used to acquire and optimize the 3D scanning data. In this case study, the 3D mesh of points was saved as an STL file and then transferred to the CATIAV5-6 2019 software to dimensionally compare the scanned 3D data and CAD model. The evaluation of the dimensional deviations was performed with the help of the deviation analysis tool within the CATIA software, where a quality control report was generated. Color comparison charts of 3D scanned model to CAD surface model which show the dimensional deviations were obtained. The dimensional deviation values of the 3D-printed molds, final molds, and CFRP specimens were determined. The sources of possible errors in the evaluation process can result from the scanning process, mesh processing, or the alignment procedure of the models being compared. The results of the dimensional deviations were statistically analyzed.

## 3. Results and Discussion

### 3.1. FDM-Based Molds Evaluation

**Figure 12:**
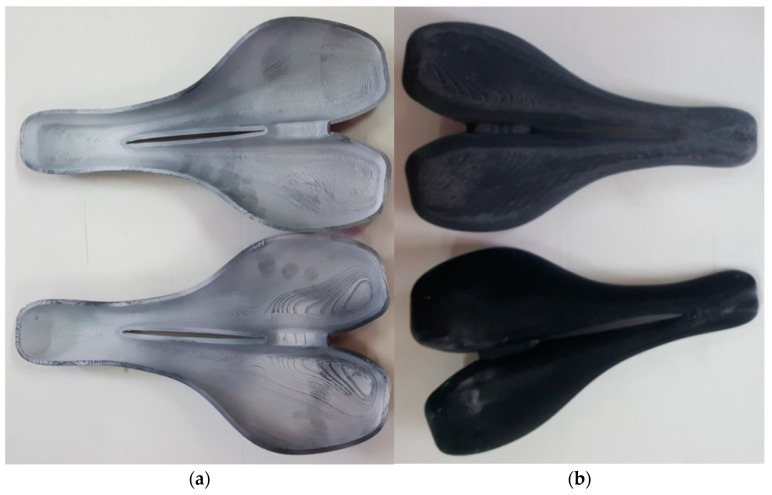
PLA mold (top) and ABS mold (down): (**a**) active surface of the molds (cavity); (**b**) back side of the molds.

Following the FDM process, two rigid molds of PLA and ABS were obtained as shown in Figure 12. The average roughness Ra of the upper mold surface was 25 ± 5 μm in both cases, as in the results obtained by Alsoufi et al. [52].

**Figure 13:**
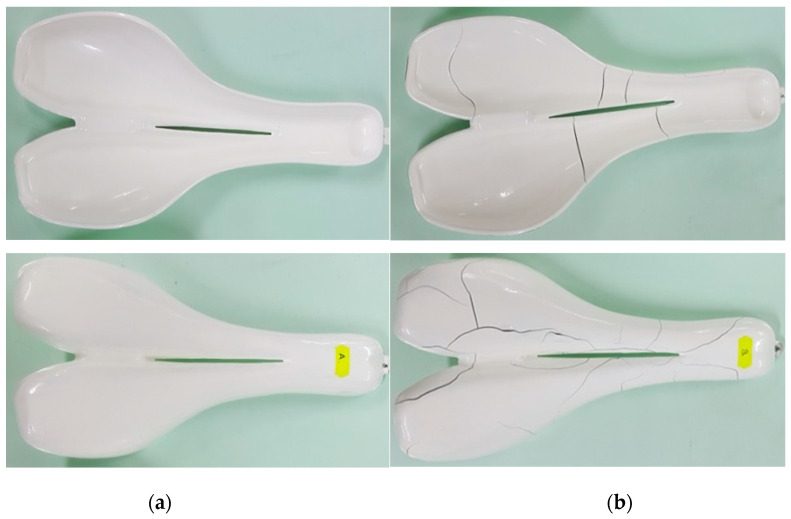
The molds after gel coat application and heat treatment: (**a**) PLA mold; (**b**) ABS mold.

The surface aspect of treated molds was analyzed. The upper and lower surfaces aspect of the treated molds is shown in Figure 13. Some cracks were detected within the gel coat layer on the surface of the ABS mold after the heat treatment, as shown in Figure 13b. Thus, the results show that the treated ABS mold cannot be used for processing laminated composite materials. The unexpected behavior of the treated ABS mold can be attributed to the heat treatment and the expansion coefficient of ABS being different than that of the gel coat, although the deformations of 3D printed ABS parts are expected to appear at over 90 °C. The PLA mold had a different behavior than the ABS mold, the gel coat layer was adherent, and no cracks or mold deformation were detected.

The surface roughness (Ra) of the PLA mold was 0.03 μm after mechanical processing and polishing. The resulted surface of the PLA mold was smooth and continuous.

The results of the comparative study between different mold manufacturing techniques showed that the PLA mold weighed 180 g, the mold from epoxy 1200 g, and the mold of aluminum 2800 g, respectively. Thus, the molds obtained by FDM have a noticeably lower total mass than the molds conventionally used. The estimated cost of the molds and detail about the estimated manufacturing time of the molds can be found in Table 3.

The estimated cost of the PLA mold was 150 euros. The PLA mold had a lower cost than the molds conventionally used, ≅10×, and ≅8.6× less in cost than CNC milled molds from the Al blocks and epoxy blocks, respectively.

The PLA molds can be heated up very easily at the same time as the laminated composite material. Due to the chosen solution, shell-type mold heating occurs rapidly. The mold surface has the same temperature as the CFRP material in the curing period time. This causes the first layer of the resin to fluidize and be pressed at the same time as the other layers. This removes the pores from the CFRP surface parts. This cannot be said of metal molds or epoxy blocks that are much harder to heat, where the outside layer is heated firstly and becomes fluid. Thus, the outside layer passes to the gel phase, seals the top layers, and the pores within the first deposited layer on the mold cannot be removed. They remain on the surface of the mold and CFRP part. It is undesirable that the surface of the CFRP specimen contains pores after the polymerization process. That generates important manual labor to cover pores and supplementary costs. From this point of view, the shell-type molds made of polymers have very good behavior.

It should also be noted that from the mechanical point of view they are quite fragile. For this reason, when the mold and CFRP are inserted into the vacuum bag and during vacuuming, the bag must be very well arranged. The bag must not strain the mold during the vacuum and curing process. It is important to leave as many as possible crimps of the bag on the surface of the mold. These can compensate for stresses generated by the vacuum pressure on both sides of the mold.

The mechanical properties of 3D printed materials by FDM (PLA and ABS) which have been post-processed by heat treatment (annealing) were studied by many authors [13,14,15]. Also, the mean ultimate strength at compression for annealed samples which were 3D printed with 100% infill was determined as 69.85 MPa [13]. The main stress of the mold during the vacuum bag-forming process and oven polymerization of CFRP is compression. The applied pressure during the CFRP part polymerization process is 0.09 MPa. In the most disadvantaged cases, this value is much lower than the compressive strength of the mold material (PLA in our case) which was 3D printed by FDM and thermal treatment.

### 3.2. Manufacturing of Bike Saddle Specimens of CFRP

The results have shown that the FDM-based mold made of PLA successfully withstands the manufacture of CFRP components. It has good behavior for the laminating of the composite materials. Heated and simultaneously exposed to a vacuum pressure of –0.9 bar, the mold remains rigid and can be used for the curing of CFRP prepreg in the oven.

**Figure 14:**
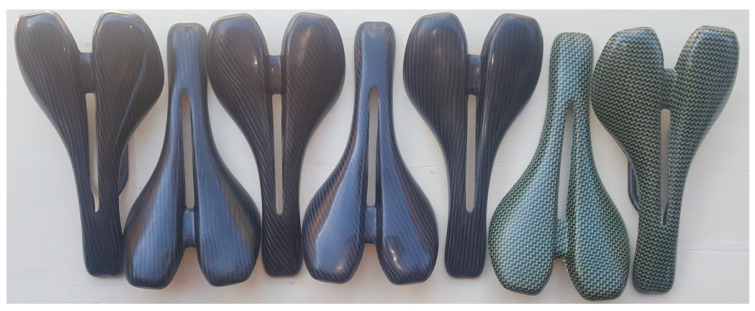
The CFRP and carbon-Kevlar fiber-reinforced polymer (CKFRP) bike saddle specimens.

Five samples from CFRP and two from CKFRP were manufactured as shown in Figure 14. A very well pressed and compact composite material of the saddle was the result. After manufacturing by oven vacuum bag curing technology of seven FRP specimens, no cracks of the gel coat layer were noticed, the mold successfully supporting the manufacture of the bike saddles.

The mass of the CFRP bike saddle specimens was 42 ± 2 g. The bike saddle assembly weight was 85 ± 2g, where two fixing bars and the structural adhesive were included supplementary.

The proposed method that uses FDM-molds is suitable for obtaining, in a rapid way, CFRP parts in limited series. Customized CFRP parts can be manufactured at a low cost compared with traditional manufacturing. However, it should be mentioned that it must be undertaken with great care, especially when the edges of the CFRP part are cut at the same dimension as the borders of the mold. The borders of the mold can deteriorate easily or the release agent layer can be damaged.

**Figure 15:**
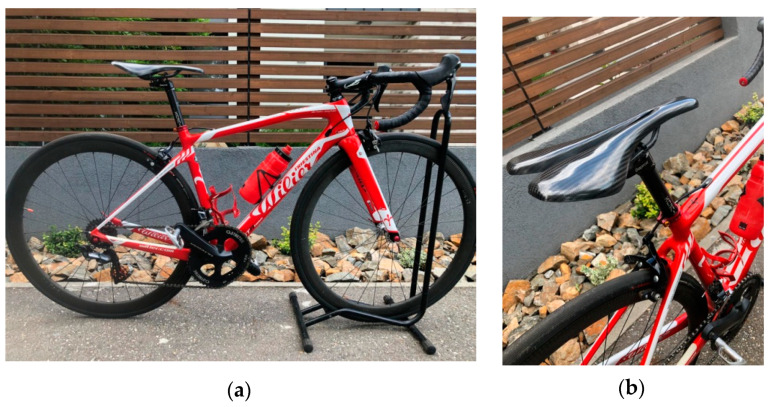
The CFRP saddle prototype assembled on a road bike: (**a**) view about the bicycle; (**b**) detail of the saddle.

The saddle assembled on a road bike (Figure 15) has resulted in a very comfortable biker position. The saddle position can be adjusted by changing the saddle tilt angle or by moving the saddle forward or backward.

### 3.3. Results of the Quality Control by 3D Scanning Technique of Molds and CFRP Parts

**Figure 16:**
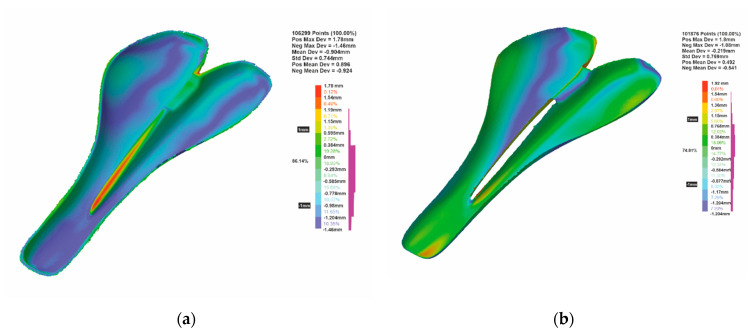
The comparison regarding the dimensional deviations from the CAD model and the FDM-based molds: (**a**) ABS mold; (**b**) PLA mold.

The molds obtained using the FDM process from ABS and PLA were dimensionally evaluated. The results indicate a mean deviation of −0.94 mm and 86.14% of the scanned points are within the ±1 mm range, in the case of the ABS mold (Figure 16a). In the case of the PLA mold, the scanning results indicate a mean deviation of −0.219 mm, and 74% of the scanned points are within the ±1 mm range (Figure 16b). These negative values indicate a contraction of the materials for these two molds. In the case of the PLA mold, the deviations of the measurements indicated that the lower limit of deformations is within acceptable limits for such parts. The heat treatment can affect the dimensional tolerances of FDM-printed parts (PLA or ABS), as was mentioned by [14]. Therefore, it is necessary to take the shrinkage or expansion of the material into consideration when designing molds to be printed by FDM and subjected to heat treatment afterward. Thus, the shrinkage of the material can be reduced by scaling the 3D model of the mold in the design process; however, in this case study the model was not scaled to eliminate this issue. A scaling factor can be determined by dimensionally comparing the CAD model and the final part after post-treatment, as is investigated in [53].

Although the process has very good accuracy, after the deposition of the material we measured a small contraction of the real parts. It should be noted that this contraction can be amplified/decreased during the scanning process, as it is known that each measuring process has an uncertainty associated with the equipment and method. The appearance of the obtained pieces is glossy and black, due to which during the scanning process a series of errors can appear, and the points taken by mistake in the air must be removed. In some scans, there were areas where the scanned points are obviously erroneous due to the geometry (the part is very thin) and the appearance of the part. In Figure 16 it can be seen that a small percentage of points is marked with red, which are positioned on the side of the saddle that theoretically cannot be deformed only in that area.

**Figure 17:**
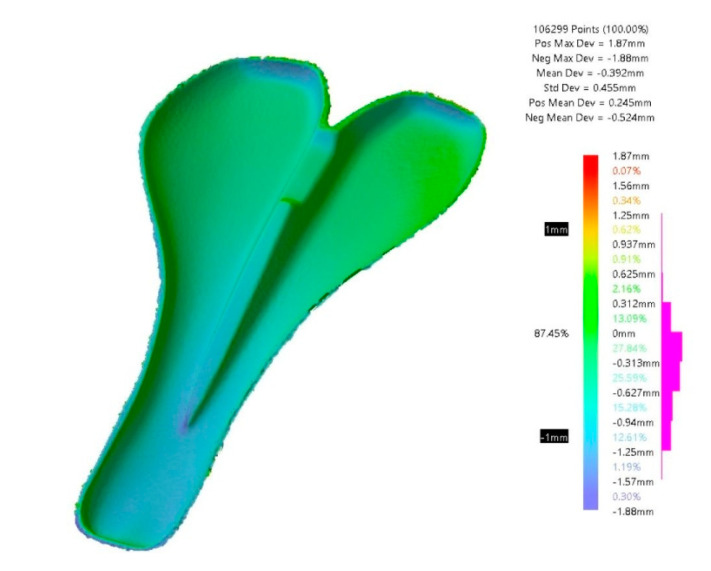
Dimensional deviation between the CAD model and the gel-coated PLA mold.

The dimensional deviations between the final PLA mold and the CAD model are shown in Figure 17. The mean deviation is −0.33 mm. The new contraction is due to the layers of gel coat applied on the mold. In this case, 87.45% of the scanned points are between ±1 mm. The value decreases from −0.21 mm to −0.33 mm regarding the mean deviation.

**Figure 18:**
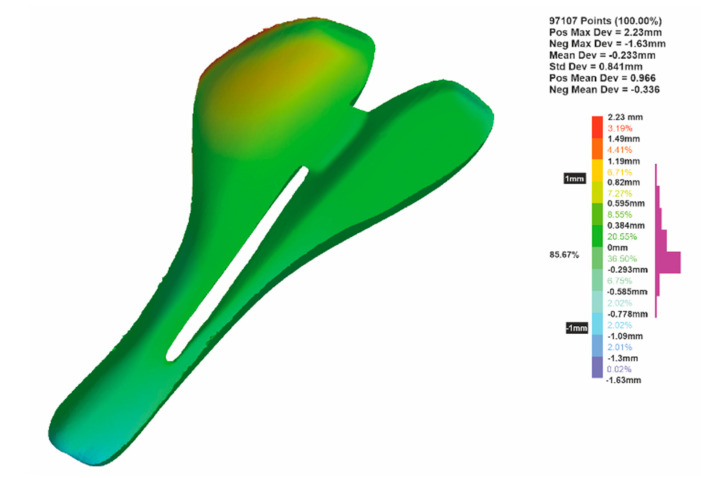
Dimensional deviation between a CFRP bike saddle specimen (A2) and the final PLA mold.

The deviations analysis between the CFRP saddle and the mold from PLA for A2 specimen is shown in Figure 18. It is found that 85.67% of the scanned points on the surface of the composite parts are between ±1 mm from the surface of the mold. In this case, the mean deviation is −0.233 mm. Similar analyzes were performed for all the specimens and the results are summarized in Table 4. The values of the mean deviation are in the range of −0.356 mm and −0.0296 mm.

**Figure 19:**
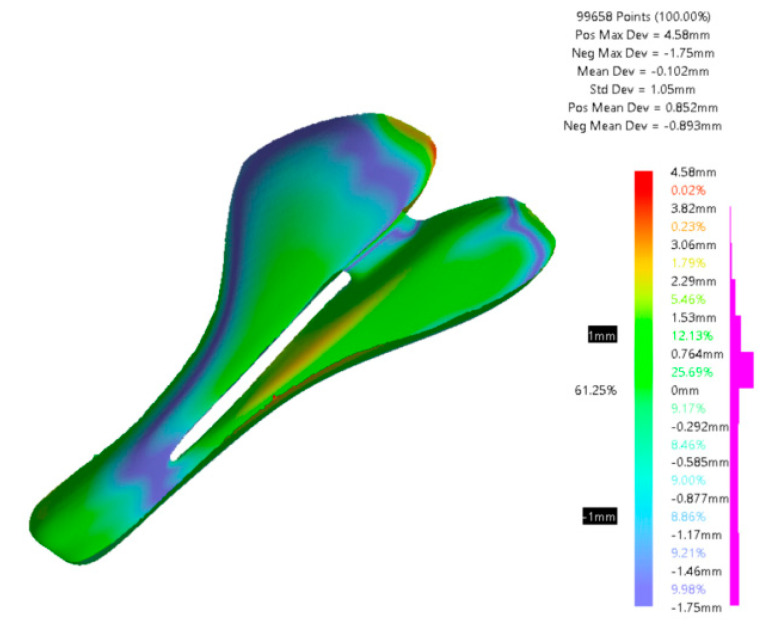
Comparison between the CAD model and specimen A3 obtained through CFRP.

A comparison between the designed CAD surface and the CFRP bike saddle is shown in Figure 19. It is found that 61% of the scanned points on the bike saddle’s surface are in the range of ±1 mm from the surface of the CAD model. Some scanning deficiencies can be noticed here as well. Part of the scanned points is also in the area of the scanned surface and another part is on the edges of the workpiece. The mean deviation, in this case, is −0.102 from the CAD model.

Detailed results of the comparison between the CFRP bike saddle and the designed CAD model for all the specimens are shown in Table 5. The values of the mean deviation are in the range of −0.349 and −0.102 mm. The results of the statistical analysis for all the five specimens are shown in Table 5.

**Figure 20:**
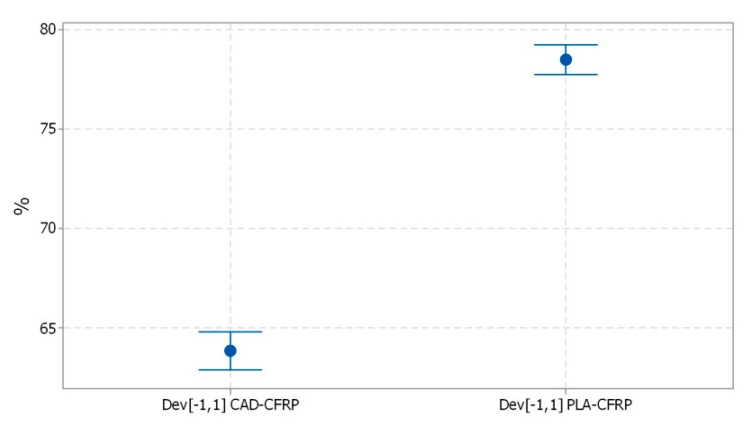
Interval plot of Dev[−1,1] CAD-CFRP and Dev[−1,1] PLA-CFRP; bars are one standard error from the mean.

Also, the interval plots of the main deviations in the range ±1 mm denoted as Dev[−1,1] of the active surface for all the samples is shown in Figure 20. Individual standard deviations were used to calculate the interval plot.

The results show that the coefficient of variation is lower than 10% that assures the data heterogeneity and expresses the repeatability of the experiments, as shown in Table 6.

The coefficient of variation (CV) is a measure of spread that describes the variation in the data relative to the mean.

## 4. Conclusions

This paper presented a novel method for the manufacturing of molds used to laminate complex geometry parts using CFRP prepreg. Specifically, the application this paper focused on was the production of a bike saddle for performance bicycles, where the design of a prototype bike saddle fabricated from CFRP was presented. The new mold and the CFRP prototype were created utilizing different technologies, where the mold was manufactured using additive manufacturing FDM processes and the CFRP fabricated part utilized vacuum bag-forming technologies and polymerization. This paper investigated the use of two materials (PLA and ABS) to create the molds through FDM.

The following conclusions can be drawn:

- The molds obtained by FDM from the thermoplastic polymers have a noticeably lower total mass than the molds conventionally used, ~15×, and ~6.6× less in mass than CNC milled molds from aluminum blocks and epoxy blocks, respectively.
- The weight of the manufactured CFRP bike saddle was measured to be 42 g and 85 g with the CFRP fixing bars fixed to the back of the saddle using adhesive. It was found that this allowed for a mass reduction of 3.52 times with the assumption that a typical saddle weighs around ~300 g [54].
- Due to the roughness of the active surface(s) of the mold(s) obtained through FDM, the active surface(s) were covered with additional layers of gel coat, as compared to the conventional mold(s). Following this, heat treatment of the mold(s) and the complete polymerization of the deposited layers were applied, and this contributes to the elimination of internal material stresses. The heat treatment comprised of exposing the specimens to 85 °C for 24 h. Subsequent to this, the ABS mold cracked, becoming unusable; it was noted that the thermal expansions of the two materials used, ABS and polyester gel coat, were different, which led to cracking of the polyester surface layer. However, it was observed that the PLA mold had very good behavior. Following the mechanical processing of the mold’s active surface (grinding and polishing), it was measured that the PLA mold had a mean dimensional deviation from the CAD model of −0.392 mm.
- The PLA mold was used to make seven bike saddles, five comprising CFRP and two CKFRP. A dimensional evaluation of the CFRP bike saddle was carried out, which produced consistent results. Following the evaluated measurements of the new CFRP bike saddles, the mean deviations as compared to the PLA mold used to laminate were in the range of –0.0838 mm and –0.38 mm. After scanning and the processing of results, it was found that over 80% of the scanned points, from the surface of the CFRP saddles (101876 scans points), are within the range of ±1 mm, compared with PLA mold. With regard to differences between CAD model and the produced parts, the mean deviation was found to be −0.3 mm, where 65% percentage of the scanned points of the bike saddle are within the range ±1 mm from the original CAD model. However, it should be noted that this is a product with a complex geometry, and a maximum size of 270 mm long and 145 mm wide.
- When evaluating the measurements for the produced CFRP bike saddle, the observed extreme values were also taken into account for evaluation. These were determined to be scan errors, creating red points on the scanning diagrams or on areas that have unscanned red points. However, it should be noted that certain areas on the edges of the saddle should not be taken into account.
- The FDM-based mold made of PLA can be used in the manufacture of parts from CFRP prepreg, utilizing vacuum bag technologies, followed by curing in an oven. However, it is recommended that these molds are to be used only in the manufacture of limited series of prototypes or customized parts.
- An important aspect of the vacuum bag process is the distribution of the vacuum bag over the mold surface whilst a vacuum is being created, where a uniform pressure on both surfaces of the mold must be applied by the vacuum bag, as this will avoid deformations which can result from a non-uniform pressure being applied to the mold, which can deform the composite and the mold during the curing process. As a result of the dimensional evaluation of the CFRP parts, deformations can only be observed on a certain part of the mold. These deformations can be attributed to where the vacuum bag deformed the mold in the central channel area of the mold. Thus, the two parts of the saddle were contracted slightly. An important conclusion with regards to the design process of the mold(s) is that it is recommended to avoid drilling holes in the central areas of the mold, and for the mold to cover the longitudinal hole with solid material, where, the mold will consolidate and the two parts can no longer come together under the action of external pressure. Following the curing process of the CFRP parts, the channel can then be machined and eliminated.
- The manufacture of CFRP parts in 3D-printed molds is a variable process that depends on parameters such as the shape and structure of the printed mold, uniform distribution of mold material, mold coating material and its thickness, constant thickness of the mold walls, uniform distribution of the vacuuming bag, vacuum pressure applied, oven baking temperature, cooling and demolding of the part.
- A future direction of research for these types of mold(s) would be to use them in the curing autoclave process of FRP. The same temperature can be used for the mold(s); however, various additional pressures that may rise up in the range of 4–5 bars will be needed. Another important study would be to investigate the design of these types of mold(s), so that they do not deform under the external pressures applied, whilst also retaining conditions resembling a low wall thickness. Additionally, studies would also need to investigate the use of different types of thermoset polymer on the mold surfaces in order to improve the stiffness and the thermal expansion qualities of the mold.

## Figures and Tables

**Table 1:** Properties of the material used in 3D printing [44,45].

Properties	PLA	ABS-M30
Density	1.24 g/cm^3^	1.04 g/cm^3^ (ASTM D792)
Tensile strength at break	53 MPa	32 MPa (ASTM D638)
Tensile elongation	6%	7% (ASTM D638)
Flexural strength	83 MPa	60 MPa (ASTM D790)
Coefficient of thermal expansion	7 × 10^−5^ mm/mm/°C	8.82 × 10^−5^ mm/mm/°C

**Table 2:** The 3D printing process parameters.

Parameters	Value for 3D Printer Leapfrog Creatr XL	Value for 3D Printer Fortus 380mc
Material	Smartfil PLA	ABS-M30 (model) SR30 (support)
Layer thickness	0.10 mm	0.178 mm
Infill	100%	100%
Print speed	70 mm/s	90 mm/s
Hotend	0.2 mm	-
Print temperature	215 °C	235 °C
Building plate temperature	70 °C	80 °C

**Table 3:** Estimate of mold cost, lead time, and the mold weight.

Manufacturing Method/Material Type	Cost	3D Print/Machining Time	Mold Weight/ Raw Material
CNC milling/Al block	1500 EUR	18 h	2800 g/6000 g
CNC milling/epoxy block	1300 EUR	14 h	1200 Kg/2600 kg
FDM process/PLA	150 EUR	18 h 37 min	180 g
FDM process/ABS	200 EUR	14 h 11 min	150 g

**Table 4:** Statistical parameters of comparison between the final PLA mold and CFRP parts.

Specimen	No. of Points	Mean Dev [mm]	Std Dev [mm]	Dev[−1,1] [%]
A1	99,412	–0.0838	0.92	78.84
A2	97,107	–0.233	0.841	85.67
A3	99,658	–0.0296	0.78	80.8
A4	100,860	–0.38	0.888	76.22
A5	101,345	–0.356	0.833	77.96

**Table 5:** Statistical parameters of comparison between mold CAD model and CFRP parts.

Specimen	No. of Points	Mean Dev [mm]	Std Dev [mm]	Dev[−1,1] [%]
A1	99,412	–0.349	0.848	66.61
A2	97,107	–0.302	0.896	64.74
A3	99,658	–0.102	0.854	61.25
A4	100,860	–0.142	0.96	64.07
A5	101,345	–0.123	0.984	62.36

**Table 6:** Statistical parameters of percentage deviations Dev[−1,1] for PLA-CFRP and CAD-CFRP for five samples.

Dev[−1,1]	Mean [%]	Std Dev [%]	CV [%]
PLA mold to CFRP parts	78.468	1.650	2.10
CAD model to CFRP parts	63.806	2.087	3.27
